# Surface ligand-regulated renal clearance of MRI/SPECT dual-modality nanoprobes for tumor imaging

**DOI:** 10.1186/s12951-024-02516-2

**Published:** 2024-05-13

**Authors:** Can Chen, Baoxing Huang, Ruru Zhang, Chaoping Sun, Lei Chen, Jianxian Ge, Dandan Zhou, Yueping Li, Shuwang Wu, Zhiyuan Qian, Jianfeng Zeng, Mingyuan Gao

**Affiliations:** 1 Center for Molecular Imaging and Nuclear Medicine, State Key Laboratory of Radiation Medicine and Protection, School for Radiological and Interdisciplinary Sciences (RAD-X), Collaborative Innovation Center of Radiological Medicine of Jiangsu Higher Education Institutions, Soochow University, Suzhou, 215123 China; 2 Clinical Translation Center of State Key Lab, The Second Affiliated Hospital of Soochow University, Suzhou, 215000 China

**Keywords:** Renal clearance, Zwitterionic ligand, Fe_3_O_4_ nanoparticles, Dual-modality imaging nanoprobe

## Abstract

**Background:**

The general sluggish clearance kinetics of functional inorganic nanoparticles tend to raise potential biosafety concerns for in vivo applications. Renal clearance is a possible elimination pathway for functional inorganic nanoparticles delivered through intravenous injection, but largely depending on the surface physical chemical properties of a given particle apart from its size and shape.

**Results:**

In this study, three small-molecule ligands that bear a diphosphonate (DP) group, but different terminal groups on the other side, *i.e.*, anionic, cationic, and zwitterionic groups, were synthesized and used to modify ultrasmall Fe_3_O_4_ nanoparticles for evaluating the surface structure-dependent renal clearance behaviors. Systematic studies suggested that the variation of the surface ligands did not significantly increase the hydrodynamic diameter of ultrasmall Fe_3_O_4_ nanoparticles, nor influence their magnetic resonance imaging (MRI) contrast enhancement effects. Among the three particle samples, Fe_3_O_4_ nanoparticle coated with zwitterionic ligands, *i.e.*, Fe_3_O_4_@DMSA, exhibited optimal renal clearance efficiency and reduced reticuloendothelial uptake. Therefore, this sample was further labeled with ^99m^Tc through the DP moieties to achieve a renal-clearable MRI/single-photon emission computed tomography (SPECT) dual-modality imaging nanoprobe. The resulting nanoprobe showed satisfactory imaging capacities in a 4T1 xenograft tumor mouse model. Furthermore, the biocompatibility of Fe_3_O_4_@DMSA was evaluated both in vitro and in vivo through safety assessment experiments.

**Conclusions:**

We believe that the current investigations offer a simple and effective strategy for constructing renal-clearable nanoparticles for precise disease diagnosis.

**Graphical Abstract:**

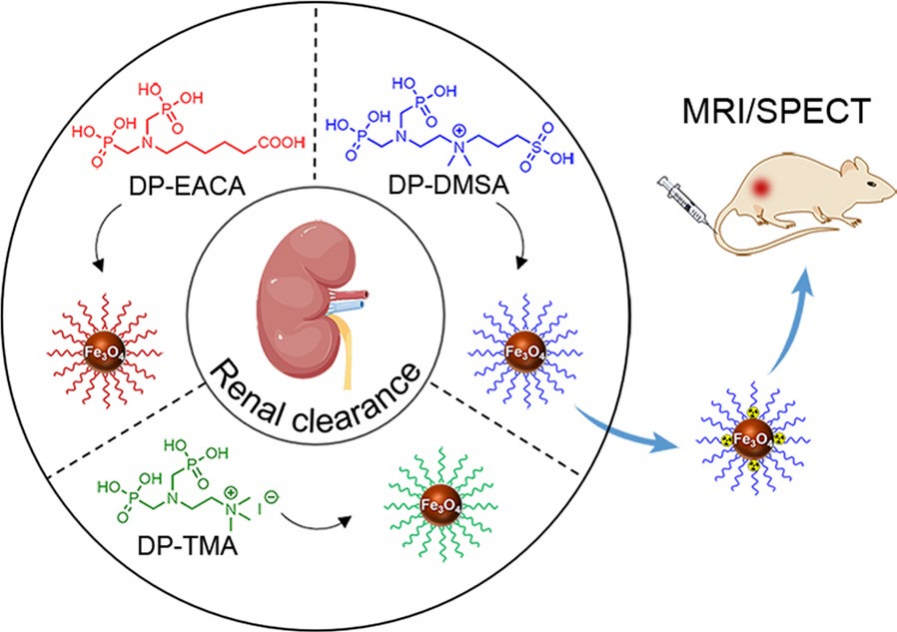

**Supplementary Information:**

The online version contains supplementary material available at 10.1186/s12951-024-02516-2.

## Introduction

With the widespread application of functional inorganic nanoparticles in the field of biomedicine, researchers have increasingly focused their attention on the biosafety of these nanoparticles—a critical prerequisite for their translation from laboratory investigation to clinic practice. Generally, the majority of functional inorganic nanoparticles tend to accumulate within the reticuloendothelial system (RES) after intravenous administration, particularly in the liver and spleen, exhibiting slow and inefficient clearance from the body [1–3]. The prolonged residence of functional inorganic nanoparticles within the body raises considerable biosafety concerns on long-term toxicity for in vivo applications [4–7]. Of particular attention, in the context of nuclear medicine applications, the extended retention of functional inorganic nanoparticles labeled radionuclides may increase the exposure of undesired radiation [8, 9]. To mitigate these potential concerns, concerted efforts have been directed toward rapid elimination and metabolism of those nanoparticles [10].

In this milieu, renal clearance emerges as a highly advantageous pathway, characterized by minimal interactions and metabolic processes within the body, as well as a faster clearance rate compared to hepatobiliary pathways [11–13]. The renal clearance pathway significantly mitigates the potential toxicity risk of nanoparticles, especially nondegradable ones composed of heavy metals ions or other toxic elements [14]. For this reason, numerous functional inorganic nanoparticles have been redesigned to facilitate renal clearance [15, 16]. The unique structure of the glomerular capillary wall plays a pivotal role in determining the renal clearance performance of nanoparticles [17]. To successfully traverse the glomerulus and undergo renal excretion, nanoparticles must navigate through three layers of the glomerular capillary wall, including the endothelial fenestrae, glomerular basement membrane, and epithelial filtration slits between podocyte processes, which is strongly associated with the size, shape, and surface physical chemical properties of the nanoparticles [11, 17–19]. Especially in terms of nanoparticle size, only nanoparticles with hydrodynamic diameter below approximately 6 nm can be more easily and effectively excreted through the kidneys owing to the constraints imposed by the pore structure of the glomerular capillary wall [11]. Hence, the precise control over the core size of nanoparticles and the judicious selection of appropriate surface ligands become critically important when designing renal-clearable inorganic nanoparticles [20]. Currently, the synthesis of ultrasmall nanoparticles with a core size less than 5 nm poses no significant challenge [21–24], but the modification of several commonly used hydrophilic surface ligands tends to considerably increase the hydrodynamic size of nanoparticles. This effect is particularly pronounced in the case of hydrophilic macromolecules, *e.g.*, polyethylene glycol (PEG) and polyvinylpyrrolidone (PVP), which often fall to meet the stringent size criteria for renal clearance [25–27]. In contrast, small-molecule ligands yield significantly smaller hydrodynamic size for nanoparticles compared to hydrophilic macromolecules [28–33]. For instance, Wei et al. successfully employed a zwitterionic dopamine sulfonate ligand to modify ultrasmall Fe_3_O_4_ nanoparticles with a core size of 3 nm, thereby achieving a hydrodynamic size of 4.7 nm conducive to renal clearance [34]. Inspired by these findings, we envision that the development of renal-clearable functional inorganic nanoparticles based on the integration of ultrasmall nanoparticles and small-molecule ligands holds immense promise for realizing excellent biocompatibility in biomedical applications.

In addition, the surface physical chemical properties of nanoparticles, especially the surface charge, also play a non-negligible role in renal filtration due to the negative charged glomerular capillary wall [11]. Commonly, cationic nanoparticles were observed to be more easily cleared by the kidneys than anionic nanoparticles and it was believed that the electrostatic repulsion of the glomerular capillary wall slowed down the renal clearance of anionic nanoparticles [17]. Balogh et al. reported a higher excretion of the 5-nm positively charged gold-composite nanodevices (Au-CNDs) in urine (33.41% ID) compared to that of the 5-nm negatively and neutrally charged Au-CNDs (8.07% ID and 7.97% ID, respectively) [35]. But some studies have also reported that nanoparticles with strong negative charge was cleared faster by the kidneys than those with weak negative charge [36], and may even be faster than those with positive charge [37]. For example, Ning et al. reported that the glycine-cysteine coated gold nanoparticles (Gly-Cys-AuNPs) with hydrodynamic size of 3.12 nm and zeta potential of -27.33 mV exhibited a higher renal clearance efficiency compared to Cys-AuNPs with hydrodynamic size of 2.69 nm and zeta potential of -12.52 mV [36]. The renal clearance efficiencies of Gly-Cys-AuNPs and Cys-AuNPs were around 41.6% and 21.5% ID, respectively, 24 h post-injection. Kang et al. reported that although all hydrodynamic size were smaller than 5.5 nm, urinary excretion of negatively charged nanocarriers (ZW800-CDPL^−^) exceeded 80% ID 4 h post-injection, whereas only approximately 45% ID was observed for positively charged ones (ZW800-CDPL^+^). As these results contradict each other, further exploration is needed to reveal the effect of surface charge on the renal clearance for optimizing functional inorganic nanoparticles towards biomedical applications.

Herein, we designed and synthesized three small-molecule ligands with different terminal groups (anion, cation, and zwitterion) for capping ultrasmall Fe_3_O_4_ nanoparticles. Our objective was to investigate the surface ligand-regulated renal clearance behaviors of ultrasmall nanoparticles. Additionally, based on the ultrasmall Fe_3_O_4_ nanoparticles with optimal renal clearance, a nanoprobe capable of conducting dual modality imaging with magnetic resonance imaging (MRI)/nuclear medicine imaging (NMI) was constructed to reveal its potential application in early tumor diagnosis. The biocompatibility of these nanoparticles was also accessed in vitro and in vivo. The current studies on renal clearance behavior regulated by surface ligands not only help to optimize the structure of renal-clearable nanoparticles but also provide guidance for their potential applications.

### Experimental section

#### Chemicals

Oleylamine, oleic acid, 1-octadecanol and 1-octadecene were purchased from Sigma-Aldrich. Dichloromethane (DCM), tetrahydrofuran (THF), 6-aminocaproic acid, formaldehyde, nitrosonium tetrafluoroborate (NOBF_4_), iodomethane (CH_3_I), trifluoroacetic acid (TFA), *N,N*-dimethylformamide (DMF), 1,3-propanesulfonate, phosphorous acid, *N,N*-dimethyl-1,2-ethanediamine, di-*tert*-butyl decarbonate (Boc), hydrogen peroxide (30%), stannous chloride (SnCl_2_) and cyclohexane were obtained from Shanghai Aladdin Biochemical Technology Co., Ltd. Hydrochloric acid (37%), nitric acid, acetone, ether and ethanol were sourced from Sinopharm Chemical Reagent Co., Ltd, China. Na^99m^TcO_4_ was acquired from Shanghai GMS Pharmaceutical Co., Ltd. Milli-Q water (> 18 MΩ·cm) was used throughout the experiments. All reagents were used without further purification.

#### Characterizations

^1^H NMR spectra were recorded on a Bruker Avance spectrometer (Bruker, Switzerland, AVANCE NEO 400 MHz). Molecular weights were determined by using a Waters ACQUITY QDa ESI–MS. Transmission electron microscope (TEM) images were captured by using an FEI Tecnai G2 transmission electron microscope operating at an acceleration voltage of 120 kV. The hydrodynamic size and zeta potential were measured at 25 °C with a Malvern Zetasizer Nano ZS90 equipped with a solid state He–Ne laser (λ = 633 nm).

### Synthesis of surface ligands

#### Synthetic of DP-EACA

*Synthesis of DP-EACA.* Initially, 6-aminocaproic acid (1.50 g, 11.45 mmol) and phosphorous acid (6.15 g, 75 mmol) were respectively dissolved in 25 mL of water. Subsequently, 5 mL of HCl (37%) was added dropwise at 0 °C. The resulting mixture was then heat to 90 °C with continuous stirring. Over a period of 30 min, 10 mL paraformaldehyde (40%, 144 mmol) was added drop by drop to the reaction mixture. Following the completion of the addition, the mixture was heat to 110 °C and refluxed for 4 h. The resulting solution was evaporated to dryness, yielding an oil. The obtained oil was then added into acetone to obtain a white precipitate. The precipitate was separated by filtration, washed with acetone, and subsequently dried under vacuum at room temperature to yield a white solid (3.35 g, 91.82%). Characterization of **DP-EACA**: ^1^H NMR (400 MHz, D_2_O) δ(ppm): 3.49 (d, 4H), 3.42 (m, 2H), 2.33 (t, 2H), 1.72 (m, 2H), 1.57 (m, 2H), 1.33 (m, 2H) (Fig. S1); ESI–MS: Calcd. for C_8_H_18_NO_8_P_2_^−^ [M-H]^−^ 318.06, found 318.08 (Fig. S2).

#### Synthetic of DP-TMA

Synthesis of compound** 1**. Initially, a solution of di-*tert*-butyl dicarbonate (7.85 g, 35.7 mmol) in 15 mL Cl_2_CH_2_ was added dropwise to a solution of *N,N*-dimethylethylenediamine (3.75 mL, 33.9 mmol) in 60 mL Cl_2_CH_2_ at 0 °C. The reaction mixture was then stirred at room temperature for 12 h. Afterward, the reaction mixture was sequentially washed with water (100 mL) and saturated salt water (2 × 100 mL), and the organic phase was dried with anhydrous Na_2_SO_4_ overnight. Finally, a colorless oil was obtained after removal of the solvent under reduced pressure (4.95 g, 77.56%). Characterization of compound **1**: ^1^H NMR (400 MHz, CDCl_3_) δ(ppm): 5.25 (bs, 1H), 3.20 (q, 2H), 2.39 (t, 2H), 2.27 (s, 6H), 1.44 (s, 9H) (Fig. S3).

Synthesis of compound** 2**. The synthesis of compound **2** started with the dissolution of compound **1** (2 g, 10.6 mmol) in 19 mL THF under a nitrogen atmosphere. Methyl iodide (0.9 mL, 14.4 mmol) was then added dropwise the solution at 0 ℃. The reaction mixture was stirred at 0 ℃ for 2.5 h. The resulting precipitate was obtained by filtration and washed three times with 15 mL of THF. Finally, the product was dried under vacuum at room temperature, yielding a white solid (2.5 g, 71.45%). Characterization of compound** 2**: ^1^H NMR (400 MHz, D_2_O) δ(ppm): 3.50 (m, 2H), 3.38 (t, 2H), 3.10 (s, 9H), 1.36 (s, 9H) (Fig. S4).

Synthesis of compound **3**. To a solution of compound **2** (2.5 g, 7.5 mmol) in 25 mL Cl_2_CH_2_, 2.5 mL TFA was added dropwise at 0 ℃ and stirred for 30 min. Afterward, the reaction mixture was warmed up to room temperature and stirred for additional 3 h. The resulting solid was filtered out and dried under vacuum at room temperature, yielding a yellow solid (1.14 g, 66.16%). Characterization of the compound **3**: ^1^H NMR (400 MHz, D_2_O) δ(ppm): 3.65 (m, 2H), 3.49 (m, 2H), 3.18 (s, 9H) (Fig. S5).

*Synthesis of DP-TMA.* The compound **3** (1.14 g, 5 mmol) and phosphorous acid (2.67 g, 32.5 mmol) were dissolved in the 24 mL of water. Subsequently, 2.2 mL of HCl (37%) was added dropwise at 0 ℃. The mixture was heat to 90 ℃ under continuous stirring, then 4.3 mL of paraformaldehyde (37%, 62.6 mmol) was added drop by drop to the reaction mixture within 30 min. Afterwards, the resulting mixture was heat to 110 ℃ and refluxed for 4 h. The obtained solution was cooled to room temperature and the solvent was removed under reduced pressure using a rotary evaporator. The obtained concentrated solution was then added to acetone to precipitate a white solid. The product was further purified with ion exchange resin to yield a white solid (0.19 g, 13.1%). Characterization of **DP-TMA**: ^1^H NMR (400 MHz, D_2_O) δ(ppm): 3.42 (m, 2H), 3.20 (m, 2H), 3.07 (s, 9H), 2.56 (d, 4H) (Fig. S6); ESI–MS: Calcd. for C_7_H_21_N_2_O_6_P_2_^+^ [M]^+^ 291.09, found 291.16 (Fig. S7).

#### Synthetic of DP-DMSA

Synthesis of compound **4**. The compound **1** (1.5 g, 7.97 mmol) was dissolved in 10 mL anhydrous DMF. A solution of 1,3-propanesulfonate (1.5 g, 12.3 mmol) in 2.5 mL anhydrous DMF was added dropwise to the solution within 10 min, and the mixture was stirred for 24 h. The resulting precipitate was collected and washed three times with ethyl acetate (3 × 10 mL) to obtain compound **4** as a white solid (2.07 g, 83.8%). Characterization of compound **4**: ^1^H NMR (400 MHz, CDCl_3_) δ(ppm): 6.05 (m, 2H), 5.99 (m, 2H), 5.95 (m, 2H), 5.64 (s, 6H), 5.46 (t, 2H), 4.72 (m, 2H), 3.92 (s, 9H) (Fig. S8).

Synthesis of compound **5**. To a solution of compound **4** (2.07 g, 6.67 mmol) in 5 mL Cl_2_CH_2_, 5 mL TFA was added dropwise at 0 ℃ and stirred for 30 min. Afterward, the reaction mixture was warmed up to room temperature and stirred for additional 3 h. The solvent was removed under reduced pressure using a rotary evaporator, resulting in an oil. The oil was then subjected to purification with methanol and ether. A white solid was obtained by filtration and dried under vacuum at room temperature, yielding a white solid (2.03 g, ~ 100%). Characterization of compound **5**: ^1^H NMR (400 MHz, CDCl_3_) δ(ppm): 6.20 (m, 2H), 6.06 (m, 2H), 6.03 (m, 2H), 5.70 (s, 6H), 5.48 (t, 2H), 4.74 (m, 2H) (Fig. S9).

*Synthesis of DP-DMSA.* The compound **5** (2.03 g, 9.67 mmol) and phosphorous acid (4.1 g, 50 mmol) were dissolved in 34 mL of water. Then, 3.33 mL of HCl (37%) was added dropwise at 0 ℃. The mixture was heat to 90 ℃ under continuous stirring, and then 10.5 mL paraformaldehyde (37%, 140 mmol) was added drop by drop to the reaction mixture within 30 min. At the end of the addition, the mixture was heat to 110 ℃ and refluxed for 4 h. Finally, the resulting solution was evaporated to dryness, resulting in an oil. Subsequently, the obtained oil was purified with ethanol and ion exchange resin to obtain a white solid (0.38 g, 9.9%). Characterization of **DP-DMSA**: ^1^H NMR (400 MHz, D_2_O) δ(ppm): 3.48 (t, 2H), 3.42 (m, 2H), 3.28 (t, 2H), 3.10 (s, 6H), 3.91 (t, 2H), 2.64 (d, 4H), 2.17 (m, 2H) (Fig. S10); ESI–MS: Calcd. for C_9_H_23_N_2_O_9_P_2_S^−^ [M-H]^−^ 397.06, found 397.06 (Fig. S11).

#### Synthesis of hydrophobic Fe_3_O_4_ nanoparticles

Hydrophobic Fe_3_O_4_ nanoparticles with a core size of 3.8 nm were prepared using a flow synthesis system according to previous reports with slight modification [38]. Typically, Fe (acac)_3_ (7.1 g, 20 mmol), oleic acid (38.7 g, 137 mmol), and oleylamine (36.6 g, 137 mmol) were dissolved in 900 mL methylbenzene to obtain a flow reaction solution. The flowing reaction liquid was pumped into the tubular reactor through a high-pressure constant current pump. The flow rate, reaction temperature, and residence time were set to 30 mL/min, 270 ℃, and 6 min, respectively. After the reaction, the reaction solution was cooled to room temperature and collected with sample bottles. The crude product was precipitated by acetone, collected by centrifugation, and dissolved by cyclohexane for three cycles. Finally, the resultant nanoparticles were redispersed in cyclohexane for further experiments.

### Ligand exchange to prepare hydrophilic Fe_3_O_4_ nanoparticles

DP-EACA, DP-TMA and DP-DMSA modified Fe_3_O_4_ nanoparticles (denoted as Fe_3_O_4_@EACA, Fe_3_O_4_@TMA and Fe_3_O_4_@DMSA, respectively) were obtained by the ligand exchange method. First, 3 mL of DMF and 10 mg of NOBF_4_ were introduced into 4 mL of cyclohexane containing 10 mg of hydrophobic Fe_3_O_4_ nanoparticles. The mixture was vortexed for 15 min and then centrifuged at 4000 g for 10 min. After removing the supernatant, the precipitate was dissolved in a mixed solution of DMF/anhydrous ether/cyclohexane (v/v/v = 1:2:8), followed by centrifugation at 7000 g for 10 min. The aforementioned procedures were repeated for 3 times. In the meantime, 100 mg of DP-EACA/DP-TMA/DP-DMSA was dissolved in 2 mL ultrapure water, with pH of the resulting solution adjusted to 7–8 with 10 M NaOH. Then, the resulting ligands solution was mixed with Fe_3_O_4_ nanoparticles obtained as mentioned above in a mixture of DMF and water. We used a volume ratio of H_2_O: DMF: Fe_3_O_4_: ligands solution = 3: 5: 1: 2 and conducted this reaction in a water bath overnight at 70 ℃. Afterward, the Fe_3_O_4_ nanoparticles were precipitated with acetone and dissolved in ultrapure water. Finally, the resulting nanoparticle solution was purified through ultrafiltration using 30 kDa MWCO centrifugal filter at 5000 g for 3 times, yielding the hydrophilic Fe_3_O_4_ nanoparticles for further applications.

### In vitro colloidal stability of Fe_3_O_4_ nanoparticles

The colloidal stability of the hydrophilic Fe_3_O_4_ nanoparticles was evaluated by DLS method. Specifically, the hydrodynamic sizes of the Fe_3_O_4_ nanoparticles in 1 × HEPES/PBS buffer (pH = 7.4) were monitored at various time points. This measurement protocol was repeated for a total of three cycles to ensure accuracy and consistency of the results.

### Relaxivity measurements

The relaxivity measurements were carried out on a 3.0 T preclinical MRI instrument (MRS 3000, MR Solution). A series of Fe_3_O_4_ nanoparticles aqueous solutions with different Fe concentrations in 2 mL Eppendorf tubes were prepared. The fast spin echo (FSE) sequence was used to measure T_1_ values. The T_1_ values were obtained by analysis of the MRI images with different TR values (90, 150, 300, 500, 800, 1200, 2000, 3000, 5000, and 10000 ms) and same TE value (10 ms). The multi-echo multi-slice (MEMS) images were acquired to measure T_2_ values. The T_2_ values were obtained by analysis of the MRI images with different TE values (13, 26, 39, 52, 65, and 78 ms) and same TR value (2000 ms).

### Animal

Specific pathogen free (SPF) grade BALB/c female mice were purchased from Shanghai SLAC Laboratory Animal Co., Ltd. All animal experiments reported herein were carried out according to the protocols approved by Soochow University Laboratory Animal Center. 4 mice for each group were involved in the study. The subcutaneous tumor models were established by injecting 4T1 cells (1 × 10^6^ each) into the dorsal area near the right hind limb of each mouse. BALB/c mice were anesthetized through a small animal anesthesia machine with 5% isoflurane (300 mL min^−1^) for 1 min and then anesthesia was maintained with 1.5% isoflurane (300 mL min^−1^) during the MR and SPECT/CT imaging. The body temperature keeping pad was used during the imaging to keep mice warm. The mice were euthanized by prolonged exposure to 5% isoflurane (300 mL min^−1^).

### MR imaging of animal

The experiments were conducted on a small animal MRI instrument with a magnetic field strength of 3.0 T. The mice were anesthetized with isoflurane and placed on the animal bed to collect pre-injection images at various anatomical levels (pre). Then, an aqueous solution of Fe_3_O_4_ nanoparticles (200 μL, 0.1 mmol Fe per kg body weight) was injected through the tail vein, and the MR images of different layers at different time points after injection were collected, with the mice remaining under anesthesia throughout the imaging process. The FSE sequence was performed to obtain the MR images of mice. The scan parameters were as follows: T_1_-weighted imaging: TR = 850 ms, TE = 11 ms, FOV = 40 mm × 40 mm, Thickness = 1 mm; T_2_-weighted imaging: TR = 5000 ms, TE = 68 ms, FOV = 40 mm × 40 mm, Thickness = 1 mm.

### Analysis of iron content in urine

The urine of the mice after the injection of Fe_3_O_4_ nanoparticles in 24 h was collected, and the iron content was determined by ICP–OES (Thermo Fisher Scientific iCAP 7000) after digested using a digestion solution (HNO_3_:H_2_O_2_, v/v, 2:1).

### Cellular uptake of Fe_3_O_4_ nanoparticles by macrophages

RAW 264.7 cells (1 × 10^6^ cells mL^−1^) were seeded in 6-well tissue culture plates with DMEM media (10% FBS) and grown to ~ 90% confluence. Fe_3_O_4_@EACA, Fe_3_O_4_@TMA, and Fe_3_O_4_@DMSA (100 μg/mL) were incubated with RAW 264.7 cells for 6 h at 37 ℃. Then, the medium was removed from the wells, and the cells were washed three times with PBS to eliminate nanoparticles outside of the cells. To assess the internalized iron content, a digestion solution consisting of HNO_3_ and H_2_O_2_ in a volumetric ratio of 2:1 was added to digest the cells. Following the digestion process, the Fe content within the cells was quantified using ICP–OES, providing insight into the cellular uptake of Fe_3_O_4_ nanoparticles by the macrophages.

### ^99m^Tc radiolabeling of Fe_3_O_4_@DMSA

Radioactive ^99m^Tc was labeled onto the Fe_3_O_4_@DMSA through the chelating effect of the DP groups of ligands anchoring on the particle surface. Typically, 200 μL Na^99m^TcO_4_ in saline with radioactivity of 5 mCi was firstly reduced by 20 μL stannous chloride (1 mg/mL in 0.1 M HCl) at room temperature for 5 min before adding 200 μg of Fe_3_O_4_@DMSA solution. After being gently shaken for 30 min at room temperature, the resultant ^99m^Tc-labeled Fe_3_O_4_@DMSA were purified through ultrafiltration using 30 kDa MWCO centrifugal filter at 5000 g for 3 times.

### Radiolabeling yield

The radiolabeling yield of Fe_3_O_4_ nanoparticles was caculated according to the following equation:

$$\mathrm{Radiolabeling yield}=\frac{{R}_{nanoparticles}}{{R}_{radionuclide}}\times 100\%$$

where *R*_nanoparticles_ and *R*_radionuclide_ are the radioactivities of the resultant radiolabeled Fe_3_O_4_ nanoparticles and the total radionuclide introduced, respectively.

### Radiolabeling stability assessment

To evaluate the radiolabeling stability, the purified radiolabeled nanoparticles were incubated in water and 10% FBS solution. Aliquots were extracted at time points for monitoring the radiochemical purities by ultrafiltration method.

### SPECT/CT imaging of animal

Following the injection of ^99m^Tc-labeled Fe_3_O_4_@DMSA (200 μL, 0.1 mmol Fe/kg body weight) via the tail vein into the tumor-bearing mice, SPECT/CT imaging was performed using a MILabs imaging instrument. The mice were anesthetized and positioned on the animal bed for the duration of the scan. SPECT images of the entire body were acquired at various time points after the injection, allowing for real-time monitoring of the distribution of the nanoprobe within the tumor. Throughout the course of the experiment, the mice remained under anesthesia to ensure their comfort and immobilization. Subsequently, the acquired SPECT imaging data were analyzed using the PMOD software tool.

### Cytotoxicity assay

Cytotoxicity assays were performed using both 4T1 and RAW 264.7 cells. These cells lines were cultured in DMEM supplemented with 10% and 1% Penicillin–Streptomycin at 37 ℃ in a humidified atmosphere of 5% CO_2_. 4T1 and RAW 264.7 cells were seeded at a density of 8000 cells/well (100 μL total volume/well) in 96-well plates for 24 h. Then, Fe_3_O_4_@DMSA at the indicated concentrations (0, 50, 100, 200, 400 μg/mL), were added to the cell culture medium. The cells were then incubated with these nanoparticles for an additional 24 h. To assess toxicity, 100 μL of cell culture medium containing 10 μL CCK-8 solution was added to each well of the 96-well plate and the plate was incubated in the CO_2_ incubator for additional 4 h. Enzyme dehydrogenase in living cells was oxidized by this kit to orange carapace. The quality of this product was assessed calorimetrically by using a multimode plate reader (PerkinElmer, EnSpire), with measurements based on absorbance values at 450 nm. The following formula was used to calculate the viability of cell growth:

Viability (%) = (mean absorbance value of treatment group/mean absorbance value of control group) × 100.

### Biocompatibility assessment

For the evaluation of biocompatibility, normal healthy mice were divided into two groups. One group was intravenously injected with Fe_3_O_4_@DMSA nanoparticles at a dose of 0.1 mmol Fe per kg body weight, while the other group received a saline injection for comparison. After 15 days of feeding, the mice were sacrificed, and their major organs, including the heart, liver, spleen, lungs, kidneys, and brain, were carefully collected. These harvested organs were subsequently fixed in a 4% paraformaldehyde solution and prepared for H&E staining.

### Statistical analysis

Statistical differences between mean values obtained from two sample groups were analyzed using the Student’s t-test. Asterisks (*, **, and ***) indicate the significance of p values less than 0.05, 0.01, and 0.001, respectively. The statistical analyses were carried out by GraphPad Prism v.8.

## Results and discussion

### Synthesis and characterization of small-molecule ligands

**Fig. 1:**
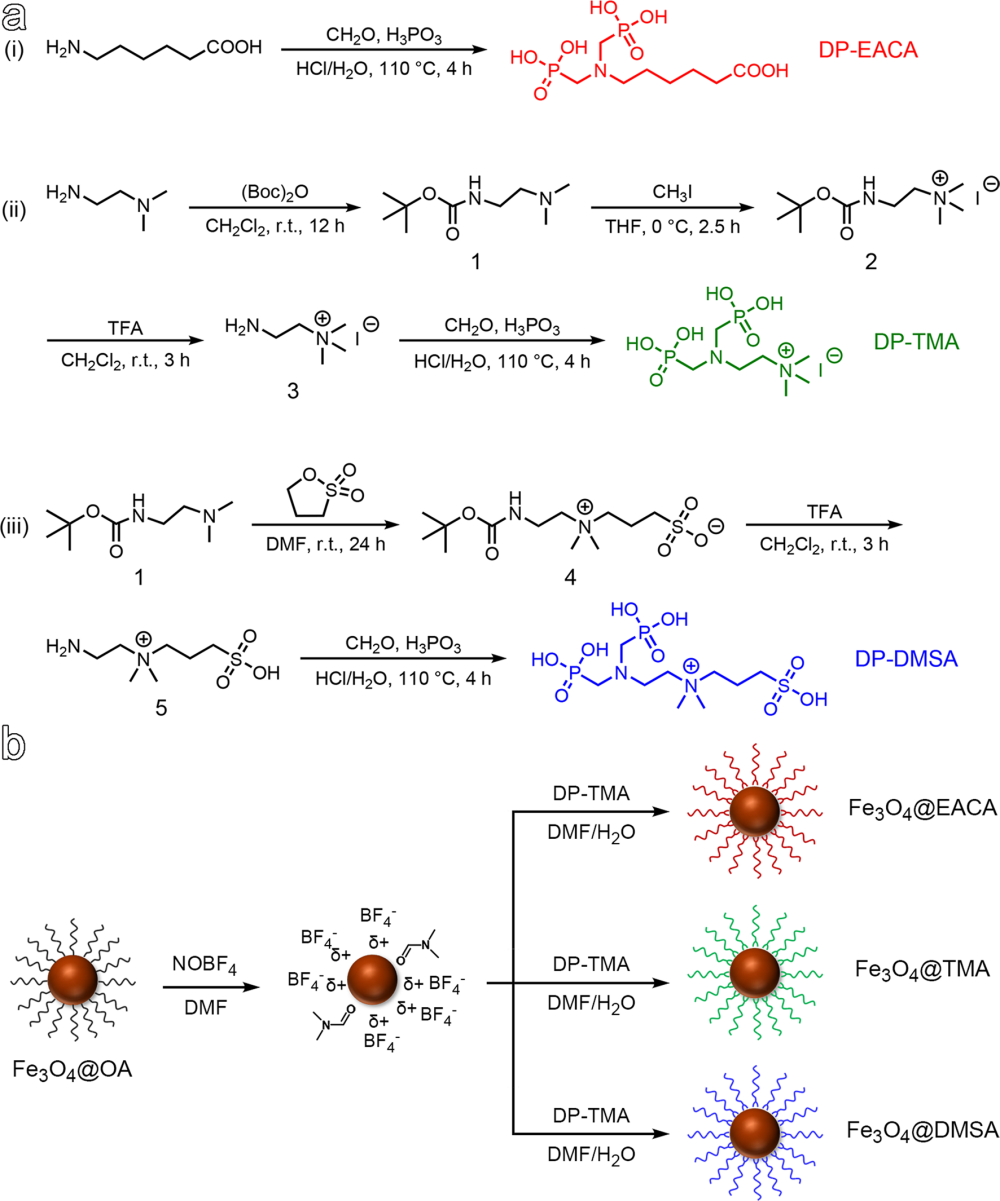
(**a**) Synthetic route of DP-EACA, DP-TMA and DP-DMSA ligands. (**b**)Schematic diagram illustrating the surface ligand modifications of Fe_3_O_4_ nanoparticles

Owing to the advantageous properties, including excellent biocompatibility and remarkable MRI performance, ultrasmall Fe_3_O_4_ nanoparticles have emerged as one of highly promising platforms for designing renal-clearable functional nanoparticles [13, 21, 39, 40]. To endow ultrasmall Fe_3_O_4_ nanoparticles with renal clearance capabilities, we designed and synthesized three distinct small-molecule ligands, each containing a diphosphonate (DP) group but different terminal groups on the other side, *i.e.*, DP-EACA, DP-TMA, and DP-DMSA, as illustrated in Fig. 1. The DP group plays a crucial role as it serves as an anchoring group for immobilizing differently charged moieties onto the surface of inorganic nanoparticles [41, 42]. Among these three ligands, DP-EACA terminated with a carboxyl group can impart an abundance of negative surface charges and enable further modification through amide condensation reactions, DP-TMA terminated with a quaternary amine group can introduce distinctive positively charged moieties to particle surface, while DP-DMSA belongs to the category of zwitterionic ligands owing to the simultaneous presence of quaternary amine and sulfonate on the other side of the ligand. Notably, zwitterionic surface is renowned as an antifouling coating with the exceptional ability to resist protein adsorption [43]. The synthetic procedures for all these three ligands are given in Fig. 1a with detailed characterization of intermediate and final products by ^1^H-NMR and ESI–MS provided in Fig. S1-11. All these results supported that the three distinct types of small-molecule ligands were successfully synthesized.

### Preparation and characterization of hydrophilic Fe_3_O_4_ nanoparticles

Ultrasmall hydrophobic Fe_3_O_4_ nanoparticles were synthesized using a flow synthesis method based on the thermal decomposition of ferric acetylacetonate according to a previous report [22]. According to the transmission electron microscopy (TEM) images and the corresponding nanoparticle size distribution profiles, the synthesized nanoparticles were nearly monodispersed with a uniform shape, and the average size of the resulting particles was 3.8 ± 0.4 nm (Fig. S12). To impart water solubility, the hydrophobic Fe_3_O_4_ nanoparticles were modified with DP-EACA, DP-TMA, and DP-DMSA, respectively. These modifications were achieved through ligand exchange, as schematically shown in Fig. 1b [44], taking advantage of higher binding affinity of DP groups to Fe^3+^ in comparison to the original hydrophobic ligand.

**Fig. 2:**
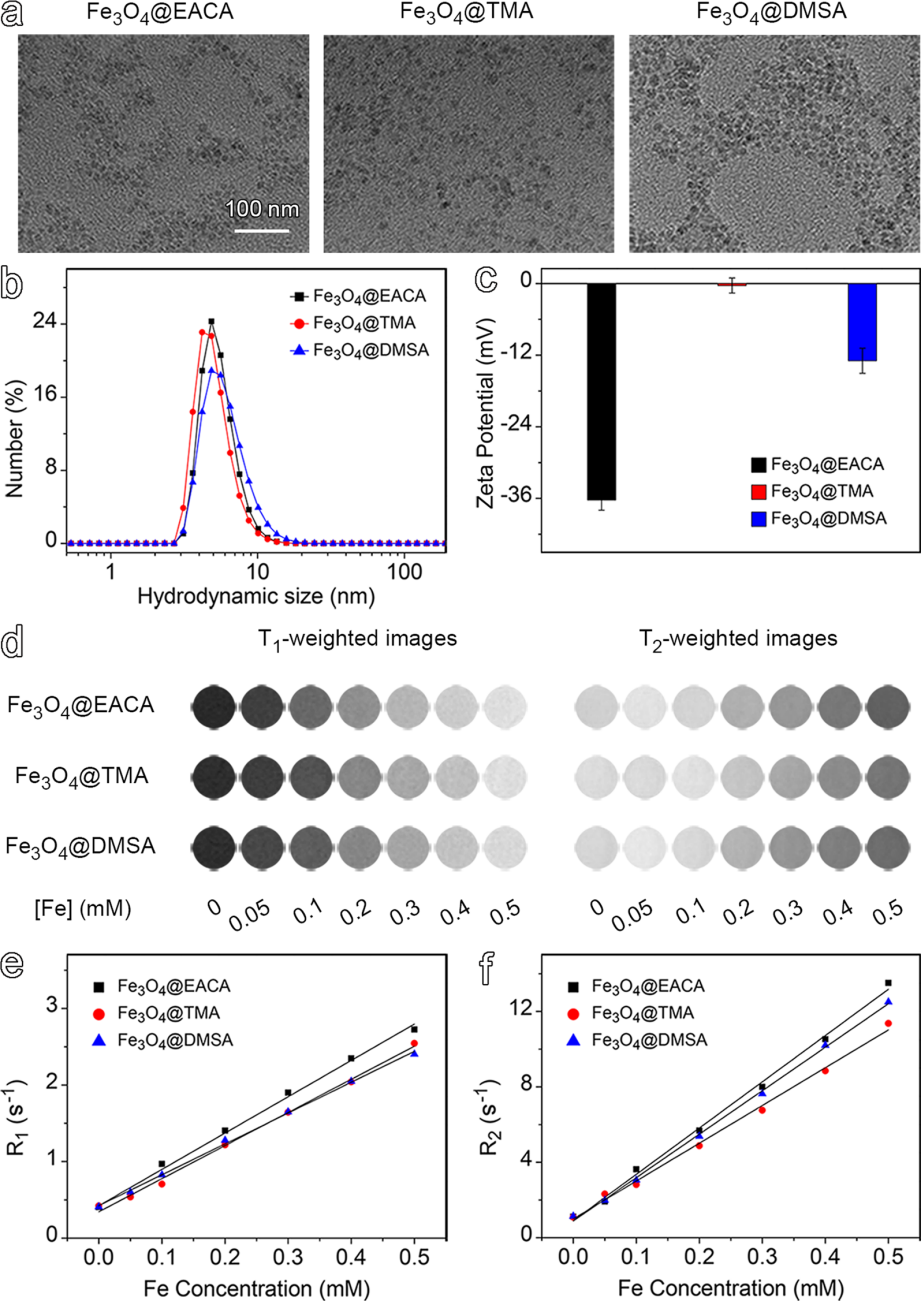
(**a**) TEM images and size distributions of hydrophilic Fe_3_O_4_ nanoparticles. Hydrodynamic size distribution (**b**) and zeta potential (**c**) of Fe_3_O_4_ nanoparticles. (**d**) T_1_- and T_2_-weighted MR images of Fe_3_O_4_ nanoparticles capped with different surface ligands. Plot of R_1_ (**e**) and R_2_ (**f**) versus Fe concentration of Fe_3_O_4_ nanoparticles

The resulting hydrophilic ultrasmall Fe_3_O_4_ nanoparticles, denoted as Fe_3_O_4_@EACA, Fe_3_O_4_@TMA, and Fe_3_O_4_@DMSA, respectively, displayed a core size and shape that remained virtually unchanged compared to their hydrophobic counterparts, as evidenced by the TEM images and size distribution profiles (Fig. 2a and Fig. S13). Dynamic light scattering (DLS) measurements reveal excellent water dispersibility with hydrodynamic size of approximately 5.4, 5.6, and 6.1 nm for Fe_3_O_4_@EACA, Fe_3_O_4_@TMA, and Fe_3_O_4_@DMSA, respectively (Fig. 2b). The DLS results demonstrated that the hydrodynamic size of the resulting particles coated with these three small-molecule ligands remained below the renal clearance threshold. Stability tests conducted over 3 days in 1 × HEPES/PBS buffer indicated negligible variations in hydrodynamic size, highlighting the excellent colloidal stability of these ultrasmall Fe_3_O_4_ nanoparticles in aqueous system (Fig. S14).

To estimate the ligand density on these ultrasmall Fe_3_O_4_ nanoparticles, thermogravimetric analysis (TGA) was employed, and the results are presented in Table S1. Approximately 1.74, 1.43, and 1.00 ligand molecules per nm^2^ were calculated for Fe_3_O_4_@EACA, Fe_3_O_4_@TMA, and Fe_3_O_4_@DMSA, respectively. The particle surface charge characteristics was determined through zeta potential measurements. As shown in Fig. 2c, Fe_3_O_4_@EACA exhibits a strong negative surface potential of -36.3 ± 1.7 mV, originating from both carboxyl groups and spare -P-O^−^ moieties of the DP anchoring groups after coordinating with Fe^3+^ ions [45]. Fe_3_O_4_@TMA displays a neutral surface potential of -0.3 ± 1.3 mV, indicating that the positive charge of the quaternary amine group are nearly neutralized by the negative charge of spare -P-O^−^ residues, which further explains the negative potential of -12.9 ± 2.1 mV for Fe_3_O_4_@DMSA, although an equivalent number of quaternary amine groups and sulfonic acid groups are co-existing on the particle surface. Therefore, it can be concluded that the DP anchoring group plays a pivotal role in the surface potential of these nanoparticles.

The MRI contrast enhancement effects of these nanoparticles were then carefully evaluated. MRI is one of the most widely used imaging techniques renowned for its ability to provide detailed anatomical information with superb spatial resolution and unlimited penetration depth, without involving ionizing radiation [46–48]. To assess the performance of these nanoparticles as MRI contrast agents, in vitro MR imaging experiments were carried out on a 3.0 T MR scanner. MR images of a series of aqueous solutions containing ultrasmall Fe_3_O_4_ nanoparticles, as given in Fig. 2d, reveal a gradual enhancement in the T_1_-weighted MR signal and a reduction in the T_2_-weighted MR signal against Fe concentration, implying that these nanoparticles are effective T_1_/T_2_ MRI contrast agents. The longitudinal relaxivity (*r*_1_) and transverse relaxivity (*r*_2_) were subsequently calculated though regression fitting of the linear correlation between the relaxation rate and Fe concentrations. The determined values were as follows: 4.74 and 24.54 mM^−1^⋅s^−1^ for Fe_3_O_4_@EACA (*r*_2_/*r*_1_ = 5.18), 4.33 and 20.03 mM^−1^⋅s^−1^ for Fe_3_O_4_@TMA (*r*_2_/*r*_1_ = 4.63), and 4.03 and 23.06 mM^−1^⋅s^−1^ for Fe_3_O_4_@DMSA (*r*_2_/*r*_1_ = 5.72), respectively (Fig. 2e, f).

### Renal clearance of surface-differently engineered Fe_3_O_4_ nanoparticles

**Fig. 3:**
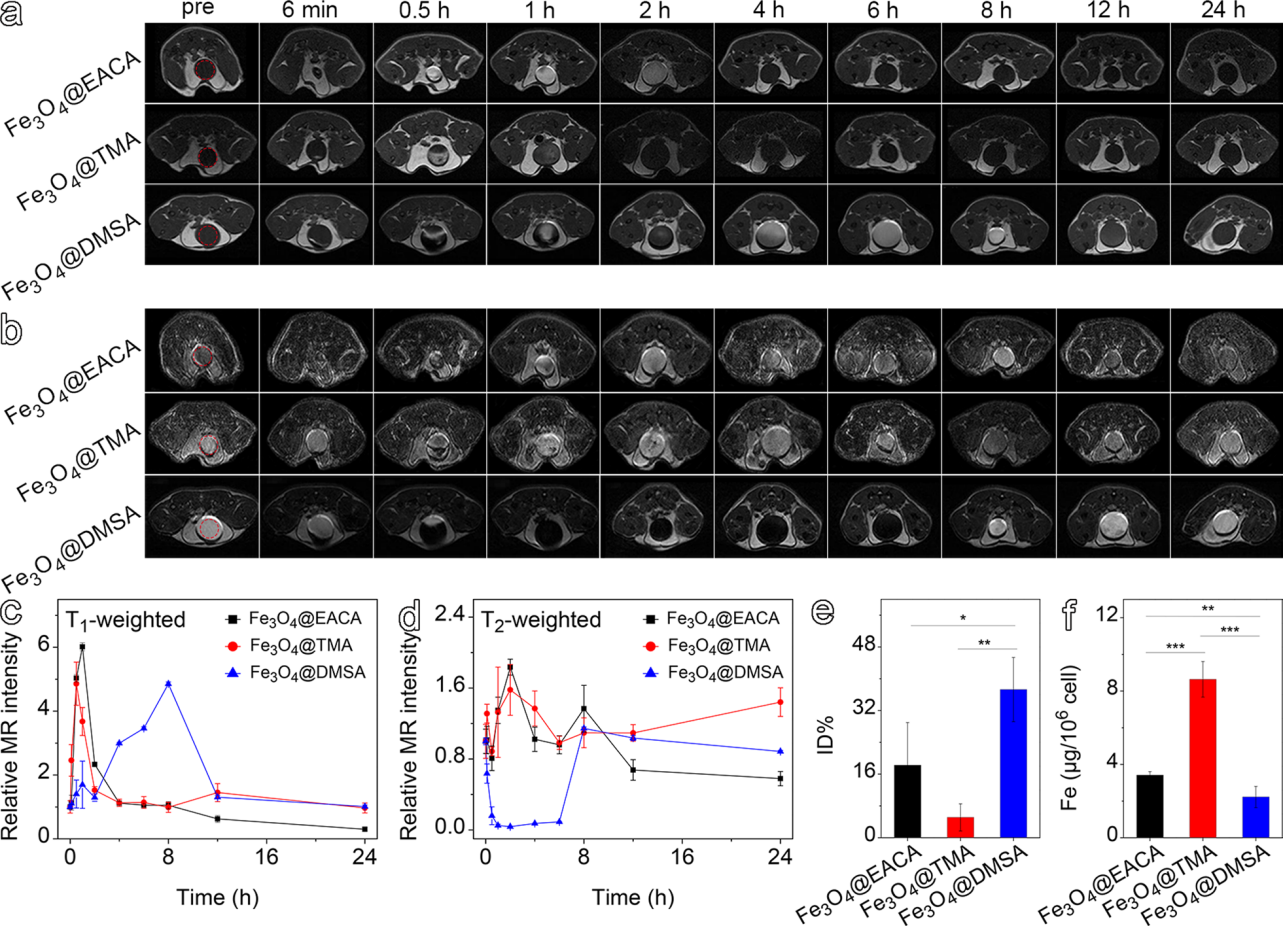
T_1_-weighted (**a**) and T_2_-weighted (**b**) MR images of bladder at different time points following injection of ultrasmall Fe_3_O_4_ nanoparticles. Temporal evolution of MR signals of the bladder for T_1_-weighted (**c**) and T_2_-weighted (**d**) imaging. (**e**) Quantification of Fe content in urine using ICP-OES. (**f)** Quantification of Fe_3_O_4_ nanoparticle internalization in RAW264.7 macrophages with different surface modifications

Since the hydrodynamic size of these three ultrasmall Fe_3_O_4_ nanoparticles are below the renal clearance threshold, it is interesting to further reveal the surface potential-associated renal clearance performance. To this end, healthy BALB/c mice were adopted as a model. After intravenous administration of the nanoparticles at a dose of 0.1 mmol Fe per kg body weight, T_1_- and T_2_-weighted MR images of the bladder section were acquired at various time points, *i.e.*, 0.1, 0.5, 1, 2, 4, 6, 8, 12, and 24 h, respectively. As shown in Fig. 3a, the T_1_-weighted MR images of bladder exhibit distinct patterns for the three ultrasmall Fe_3_O_4_ nanoparticles. In the case of Fe_3_O_4_@EACA, the bladder exhibits hyperintense signal between 0.5 and 2 h postinjection, indicative of Fe_3_O_4_@EACA clearance through kidney. Similarly, Fe_3_O_4_@TMA give rise to hyperintense signal in bladder around 0.5–1 h postinjection. Strikingly, Fe_3_O_4_@DMSA exhibits hypointense signal around 0.5 h and 2 h postinjection, followed by long-lasting hyperintense signal around 1 h and 4–8 h postinjection, respectively. Previous studies suggest that a high concentration of ultrasmall Fe_3_O_4_ nanoparticles can lead to signal attenuation during T_1_-weighted imaging [49]. Therefore, the above observations indicate that Fe_3_O_4_@DMSA is more heavily cleared through kidneys than the other two samples, which can find supportive evidence from T_2_-weighted imaging results. As shown in Fig. 3b, only a small part of bladders of mice receiving Fe_3_O_4_@EACA and Fe_3_O_4_@TMA, respectively, exhibits hypointense signal around 0.5–1 h postinjection, while the entire bladder of the mouse receiving Fe_3_O_4_@DMSA presents significant hyperintense signal from 0.5 to 6 h postinjection, supporting the favorable clearance of Fe_3_O_4_@DMSA through the kidneys. Temporal T_1_ and T_2_ signal variations in the bladders are quantitatively presented in Fig. 3c, d. With respect to Fe_3_O_4_@EACA and Fe_3_O_4_@TMA groups, the T_1_ signals are firstly increased and then decreased to show peaks around 1 h postinjection, while the T_2_ signals are slightly varied. Regarding Fe_3_O_4_@DMSA group, the enhanced T_1_ signals appear from 2–8 h postinjection, accompanied by a decreased T_2_ signal before 6 h, suggesting continuous renal clearance of Fe_3_O_4_@DMSA over an extended period. These results indicate that Fe_3_O_4_@DMSA can be cleared more extensively through the kidneys than Fe_3_O_4_@EACA and Fe_3_O_4_@TMA.

Subsequently, the amounts of ultrasmall Fe_3_O_4_ nanoparticles cleared through the kidneys were quantitatively determined by collecting urine from mice injected with the three types of ultrasmall Fe_3_O_4_ nanoparticles over a 24 h period for Fe content measurements with inductively coupled plasma-optical emission spectrometry (ICP-OES). As shown in Fig. 3e, Fe_3_O_4_@EACA, Fe_3_O_4_@TMA, and Fe_3_O_4_@DMSA are excreted by approximately 18.2% ID, 5.1% ID, and 37.3% ID, respectively, 24 h after intravenous injection. The clearance of nanoparticles from the bloodstream typically occurs through two primary pathways: uptake by the RES or clearance via the kidneys [11]. The lower renal clearance rates for Fe_3_O_4_@EACA and Fe_3_O_4_@TMA may result from their heavier phagocytosis by the RES (Fig. S15). To confirm this hypothesis, we further analyzed the uptake of ultrasmall Fe_3_O_4_ nanoparticles by RAW264.7 cells. The studies in Fig. 3f reveal that Fe_3_O_4_@DMSA has the lowest cellular uptake among all three samples, well consistent with the renal clearance results.

Despite of similar hydrodynamic sizes, the three types of ultrasmall Fe_3_O_4_ nanoparticles exhibit significant differences in renal clearance and cellular internalization. This phenomenon can be explained by the formation of protein corona, a plasma protein coating that forms when nanoparticles enter the bloodstream [50]. The protein corona alters the original characteristics of nanoparticles and significantly influences their fate, cellular internalization, biological distribution, and targeting efficacy, apart from the cytotoxicity and immune effects [51–53]. Surface ligands are known to be a crucial factor influencing the formation of the protein corona on nanoparticles [54, 55]. Therefore, it is reasonably to believe that the current ultrasmall Fe_3_O_4_ nanoparticles with different physicochemical properties should bear different protein coronas that regulate their interactions with macrophages [56]. Zwitterionic ligands, known for their high water affinity, have been reported to effectively suppress protein corona formation on nanoparticles [43, 57, 58], which explains that Fe_3_O_4_@DMSA presents the highest renal clearance rate due to the suppressed protein adsorption and consequently lowered macrophage uptake.

### In vivo MRI/SPECT dual-modal imaging of tumors

**Fig. 4:**
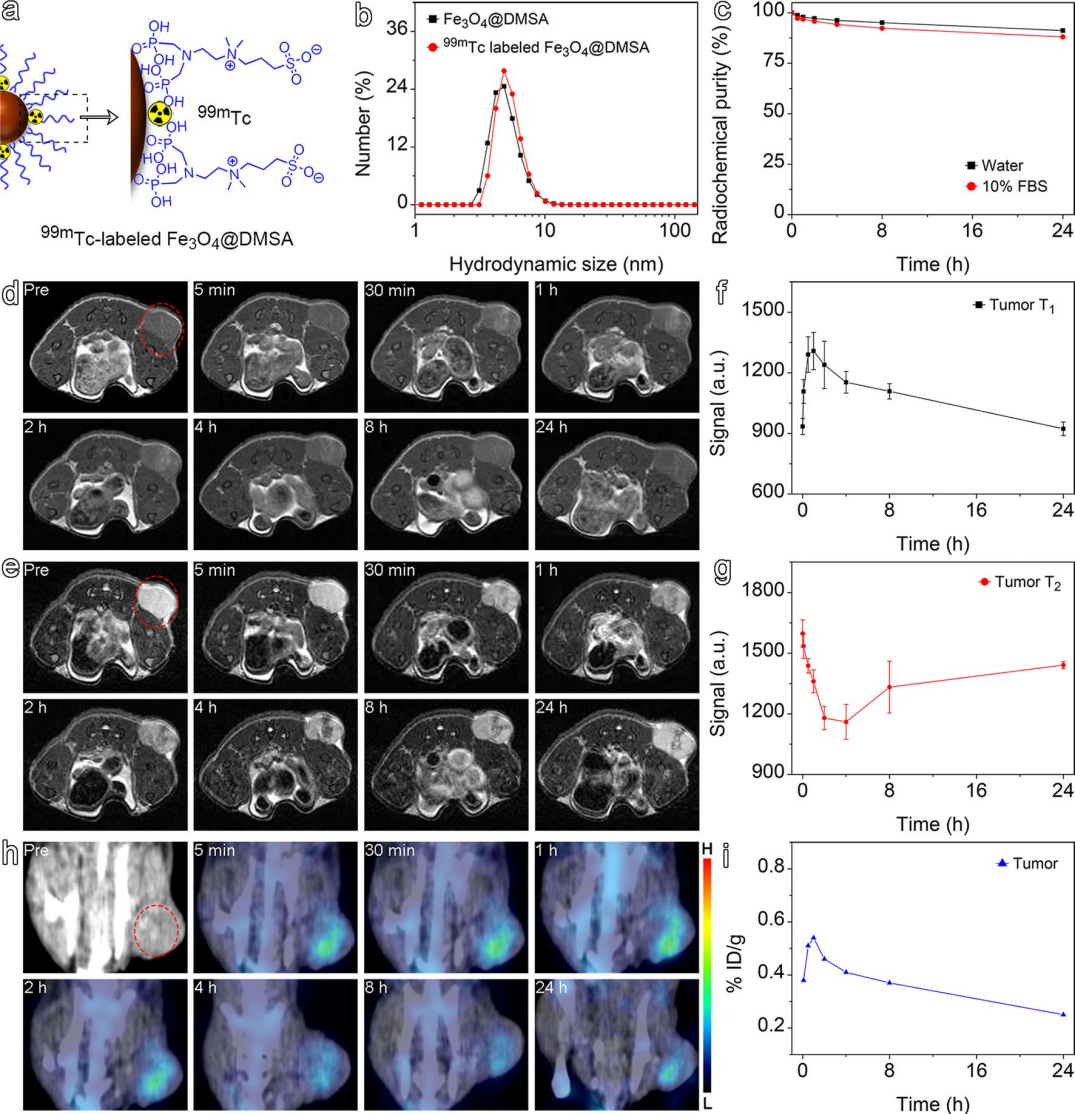
**(a)** Schematic diagram showing the structure of ^99m^Tc-labeled Fe_3_O_4_@DMSA nanoprobe. (**b)** Hydrodynamic size profiles of Fe_3_O_4_@DMSA recorded before and after ^99m^Tc labeling. (**c**) Radiolabeling stability of ^99m^Tc-labeled Fe_3_O_4_@DMSA nanoprobe in water and 10% FBS solution. T_1_-weighted (**d**) and T_2_-weighted (**e**) MRI images of mice after injection of Fe_3_O_4_@DMSA nanoprobe, and the corresponding tumor signal value changed curve of T_1_-weighted images (**f**) and T_2_-weighted images (**g**). (**h)** SPECT/CT images of the tumor-bearing mice injected with.^99m^Tc-labeled Fe_3_O_4_@DMSA nanoprobe, together with temporal γ-signals of the tumorous areas (**i**)

Owing to the outstanding renal clearance performance, DMSA was chosen as an optimized surface coating for further expanding the biomedical applications of Fe_3_O_4_ nanoparticles. Nuclear medicine imaging, including single-photon emission computed tomography (SPECT) and positron emission tomography (PET), offers very high imaging sensitivity [59, 60]. It was previously demonstrated that the diphosphonate group not only tightly binds the current particle surface ligands to Fe_3_O_4_ nanoparticles, but also provides coordinating cites for radionuclide through ligand anchoring group-mediated radiolabeling (LAGMERAL) (Fig. 4a) [38, 41, 45]. Thus, the outstanding MRI contrast enhancement performance of the ultrasmall Fe_3_O_4_ nanoparticles can be integrated with NMI to achieve high-performance MRI/SPECT dual-modality probes. In this respect, an efficient renal clearance is vital for final clinical translation as exposure of radiation to non-target tissues and organs needs to be suppressed.

To demonstrate the feasibility of using these ultrasmall Fe_3_O_4_ nanoparticles for MRI/SPECT dual-modal imaging, Fe_3_O_4_@DMSA was adopted in the following animal experiments to detect tumors in vivo, capitalizing on the enhanced permeability and retention effect associated with tumors. ^99m^Tc is a commonly used radionuclide for SPECT, so it was used to label Fe_3_O_4_@DMSA to create an MRI/SPECT nanoprobe for tumor imaging in mice. The hydrodynamic size of the Fe_3_O_4_@DMSA nanoparticles was determined before and after ^99m^Tc labeling for confirming that radiolabeling didn’t induce unwanted aggregation (Fig. 4b). Then, the radiolabeling stability was also tested by incubating the resulting ^99m^Tc-labeled Fe_3_O_4_@DMSA nanoprobe in water and 10% FBS solution, respectively. The results given in Fig. 4c reveal that the radiochemical purity remained above 88% after 24 h.

To evaluate the imaging performance of ^99m^Tc-labeled Fe_3_O_4_@DMSA nanoprobe for both MRI and SPECT, mice bearing subcutaneous 4T1 breast tumors were adopted. T_1_-weighted and T_2_-weighted MR images obtained at different time points after intravenous injection of Fe_3_O_4_@DMSA into tumor-bearing mice are provided in Fig. 4d, e. T_1_-weighted MRI results show that a hyperintense signal appears in the tumor site 30 min after injection and reaches its intensity maximum at 1 h postinjection. As shown in Fig. 4f, the hyperintense signal remains visualizable 4 h postinjection. In T_2_-weighted MRI, the tumor site presents a hypointense signal reaching it maximum 4 h postinjection (Fig. 4g). The delayed appearance of T_2_ signal maximum may be caused by the particle aggregation that presents a stronger effect on T_2_ signal than that on T_1_ signal [61]. These MRI results demonstrate that Fe_3_O_4_@DMSA exhibits excellent in vivo tumor imaging performance, characterized by high contrast enhancement effects on both T_1_ and T_2_ imaging.

Given that NMI offers significantly higher sensitivity than MRI, SPECT imaging in a parallel group of tumor-bearing mice was carried out with ^99m^Tc-labeled Fe_3_O_4_@DMSA nanoprobe. Representative SPECT images obtained at different time points following intravenous injection of the ^99m^Tc-labeled Fe_3_O_4_@DMSA nanoprobe via the tail vein are displayed in Fig. 4h. Quite evidently, the tumor site exhibits markedly higher signals than normal tissue over a time window similar to those for T_1_ and T_2_ imaging (Fig. 4i), confirming the promising application of ^99m^Tc-labeled Fe_3_O_4_@DMSA nanoprobe in MRI/SPECT dual-modal tumor imaging.

The advantage of renal clearance in accelerating the removal of nanoparticles from the body to enhance biosafety has garnered considerable attention in the development of inorganic nanoprobes for MRI [62]. For instance, Wei et al. successfully prepared renal-clearable nanoprobes by modifying ultrasmall Fe_3_O_4_ nanoparticles with a zwitterionic dopamine sulfonate ligand, resulting in positive MRI contrast agents. [34]. However, this approach poses challenges in labeling nanoparticles with radionuclides for the integration of MRI and SPECT to improve diagnostic accuracy. Intrinsic radiolabeling, which involves embedding radionuclides into the crystal lattice of inorganic nanoparticles, may offer a viable solution [45, 60]. Wei et al. demonstrated the incorporation of ^59^Fe into the inorganic core of Fe_3_O_4_ nanoparticles. [34]. Nevertheless, the complexity of the preparation process and limitations on the types of radionuclides could hinder widespread application in constructing renal-clearable MRI/SPECT dual-mode nanoprobes. [45, 60]. In this study, we propose a simple and effective strategy for achieving renal-clearable MRI/SPECT nanoprobes by combining DP-modified zwitterionic ligands with ultrasmall Fe_3_O_4_ nanoparticles, which exhibit excellent MRI performance. Our findings demonstrate the feasibility of these nanoprobes for precise tumor diagnosis. However, the current study's reliance on the enhanced permeability and retention (EPR) effect for tumor accumulation and limitations in modifying targeting ligands for active tumor targeting highlight areas for improvement.

## Safety assessment

Safety assessment of a given nanoprobe is critical for its biomedical applications. Therefore, the cytotoxicity Fe_3_O_4_@DMSA was firstly evaluated in vitro through cell counting kit-8 (CCK-8) assay. Similar to many previous cytotoxic studies on Fe_3_O_4_ nanoparticles in cell lines, Fe_3_O_4_@DMSA presented only very slight cytotoxic effects on cell lines including 4T1 and RAW 264.7 cells (Fig. S16). To further assess the biosafety of Fe_3_O_4_@DMSA in vivo, Fe_3_O_4_@DMSA was intravenously injected into healthy mice at a dose of 0.1 mmol Fe/kg, with a saline group serving as control. The mice were sacrificed 15 d postinjection to extract major organs including the heart, liver, spleen, lung, kidney, and brain. The following hematoxylin–eosin (H&E) staining results revealed no significant differences between the mice treated with Fe_3_O_4_@DMSA and saline, respectively, strongly suggesting the outstanding biocompatibility of our nanoparticles (Fig. S17).

## Conclusions

In summary, to achieve efficient renal clearance of ultrasmall Fe_3_O_4_ nanoparticles, three small-molecule ligands bearing a diphosphonate group but different terminal groups on the other side, *i.e.*, anionic, cationic, and zwitterionic groups, were designed and successfully used to replace the hydrophobic surface ligands of ultrasmall Fe_3_O_4_ nanoparticles. Systematic studies have demonstrated that ultrasmall Fe_3_O_4_ nanoparticles modified with these surface ligands not only possess sufficiently small hydrodynamic size, enabling them to be cleared through the kidneys, but also exhibit excellent T_1_/T_2_ MRI contrast enhancement performance. Combining MRI of the bladder and urine Fe content determined through ICP-OES, we have shown that the zwitterionic ligand-coated Fe_3_O_4_ nanoparticle (Fe_3_O_4_@DMSA) exhibits superior renal clearance due to its reduced uptake by the RES. With the aid of the diphosphonate anchoring group of the hydrophilic ligand, Fe_3_O_4_@DMSA has successfully been labeled with ^99m^Tc to achieve an efficiently renal-clearable MRI/SPECT dual-modality imaging nanoprobe that presents outstanding MRI/SPECT dual-modality imaging capacities in a 4T1 tumor xenograft mouse model. Furthermore, the in vitro and in vivo biosafety assessments demonstrate the excellent biocompatibility of Fe_3_O_4_@DMSA. In conclusion, we provide a simple and effective strategy for achieving renal-clearable functional nanoparticle suitable for precise disease diagnosis.

### Supplementary Information

**Supplementary Material 1:** Additional data (Fig. S1-S17 and Table S1) associated with this article can be found in the online version.

## Data Availability

All data contained in the study are in this article.
