# Supplementary material for: Surface ligand-regulated renal clearance of MRI/SPECT dual-modality nanoprobes for tumor imaging
Source: J Nanobiotechnology. 2024 May 13;22:245. doi: 10.1186/s12951-024-02516-2 (PMC11089712; doi:10.1186/s12951-024-02516-2)
Supplement: Supplementary file 1 — Supplementary Material 1: Additional data (Fig. S1-S17 and Table S1) associated with this article can be found in the online version. [file 12951_2024_2516_MOESM1_ESM.docx]

*Supporting Information*

**Surface ligand-regulated renal clearance of MRI/SPECT dual-modality nanoprobes for tumor imaging**

Can Chen,^1†^ Baoxing Huang,^1†^ Ruru Zhang,^1^ Chaoping Sun,^1^ Lei Chen,^1^ Jianxian Ge,^1^ Dandan Zhou,^1^ Yueping Li,^1^ Shuwang Wu,^1^ Zhiyuan Qian,^1^ Jianfeng Zeng,^1*^ Mingyuan Gao^1,2 *^

^1^ Center for Molecular Imaging and Nuclear Medicine, State Key Laboratory of Radiation Medicine and Protection, School for Radiological and Interdisciplinary Sciences (RAD-X), Soochow University, Collaborative Innovation Center of Radiological Medicine of Jiangsu Higher Education Institutions, Suzhou, 215123, China

^2^ Clinical Translation Center of State Key Lab, The Second Affiliated Hospital of Soochow University, Suzhou, 215000, China


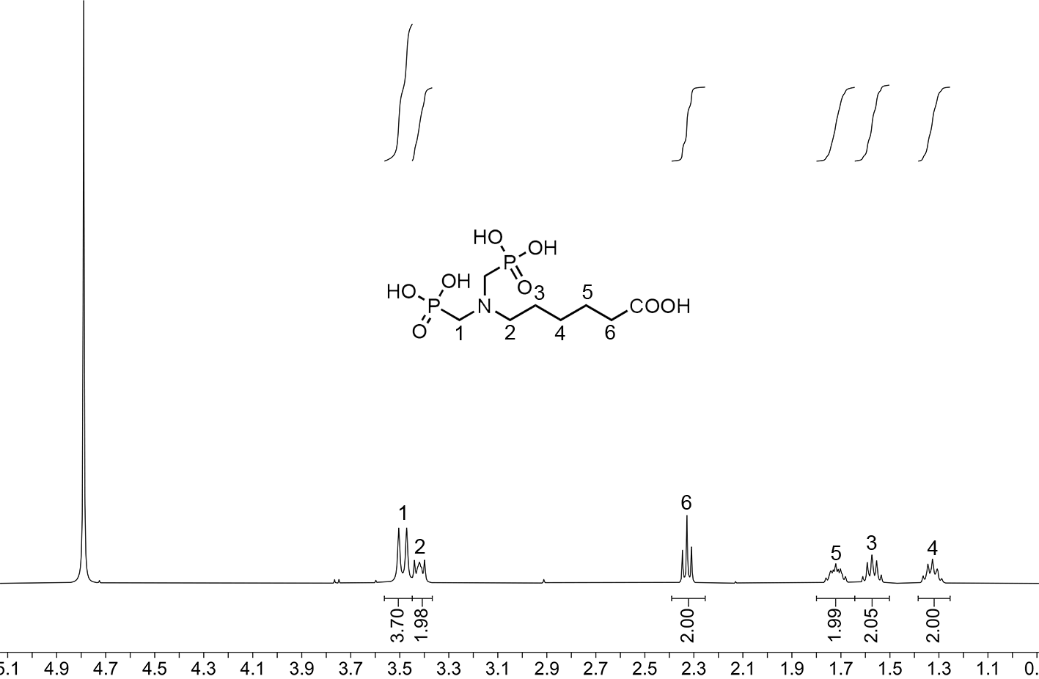


**Fig. S1** ^1^H NMR of DP-EACA.


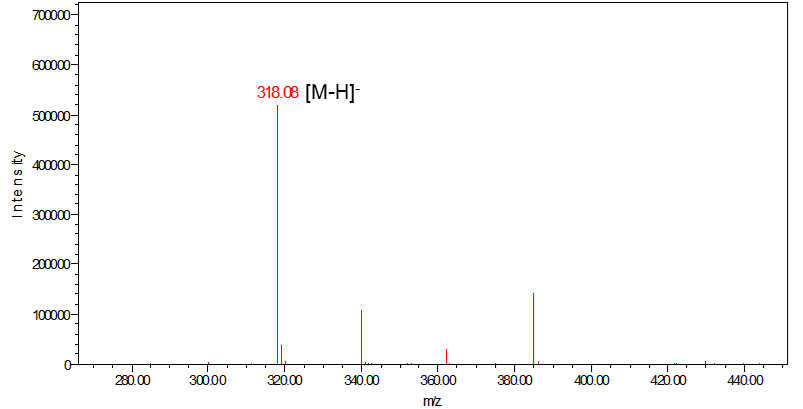


**Fig. S2** ESI-MS data of DP-EACA.


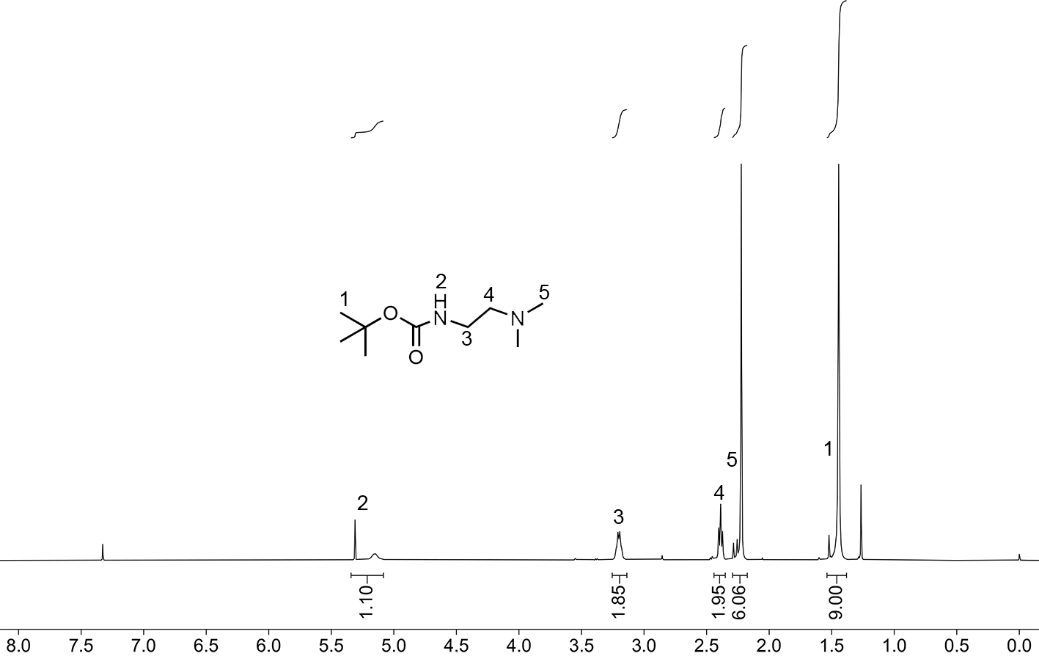


**Fig. S3** ^1^H NMR of compound **1**.


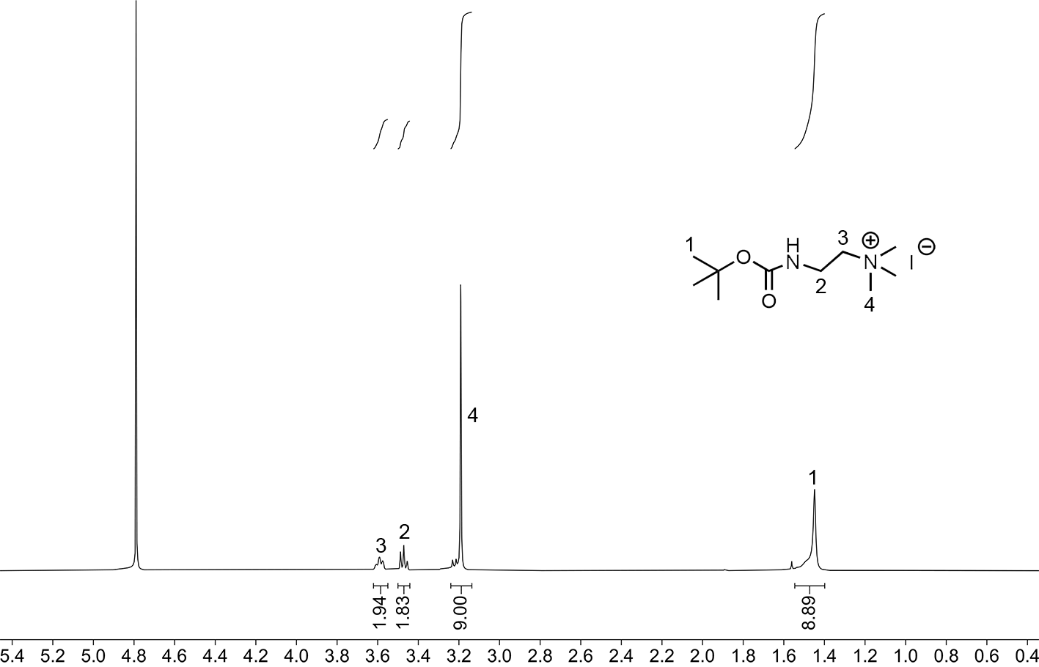


**Fig. S4** ^1^H NMR of compound **2**.


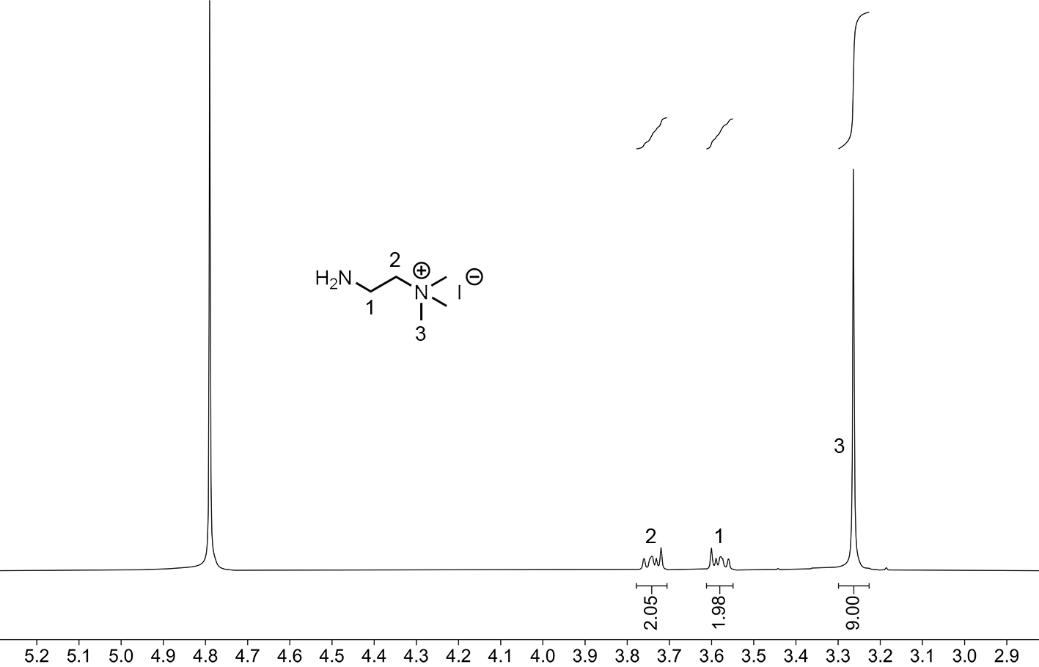


**Fig. S5** ^1^H NMR of compound **3**.


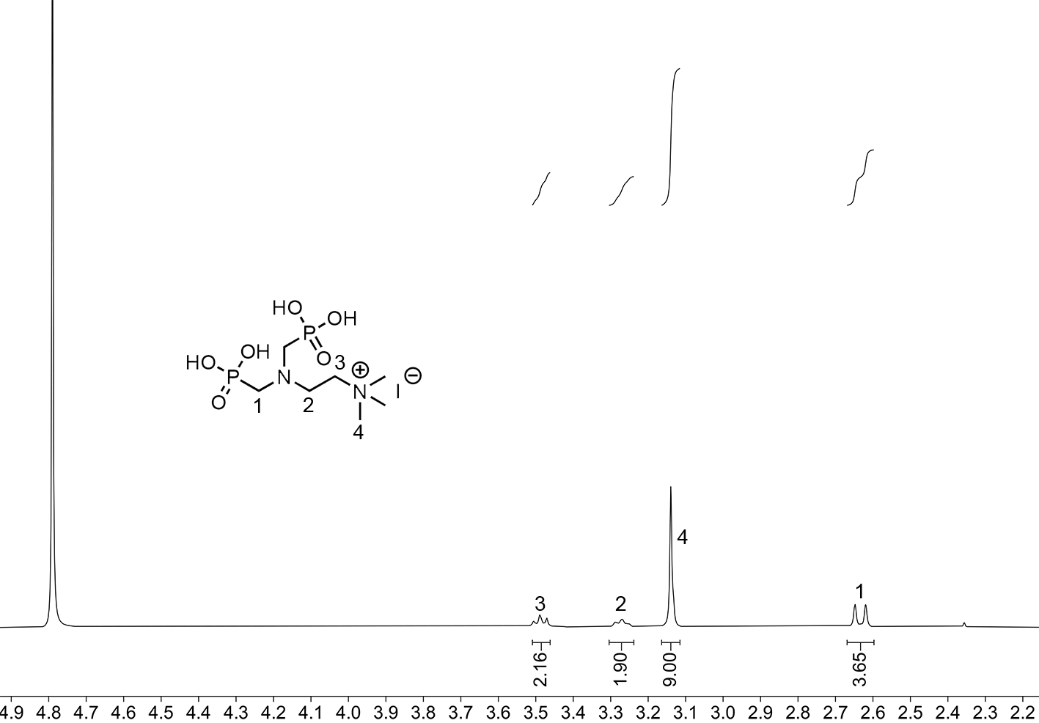


**Fig. S6** ^1^H NMR of DP-TMA.


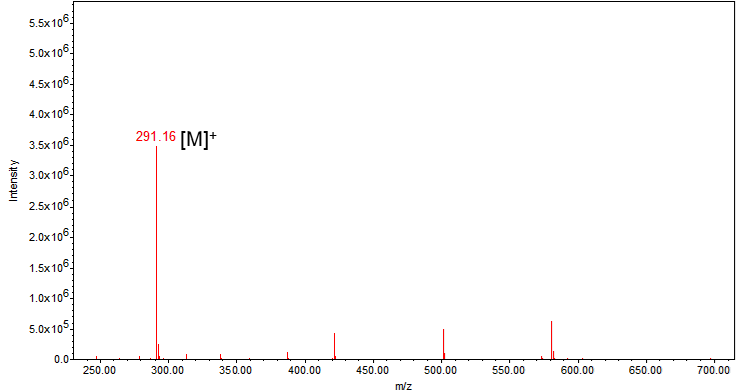


**Fig. S7** ESI-MS data of DP-TMA.


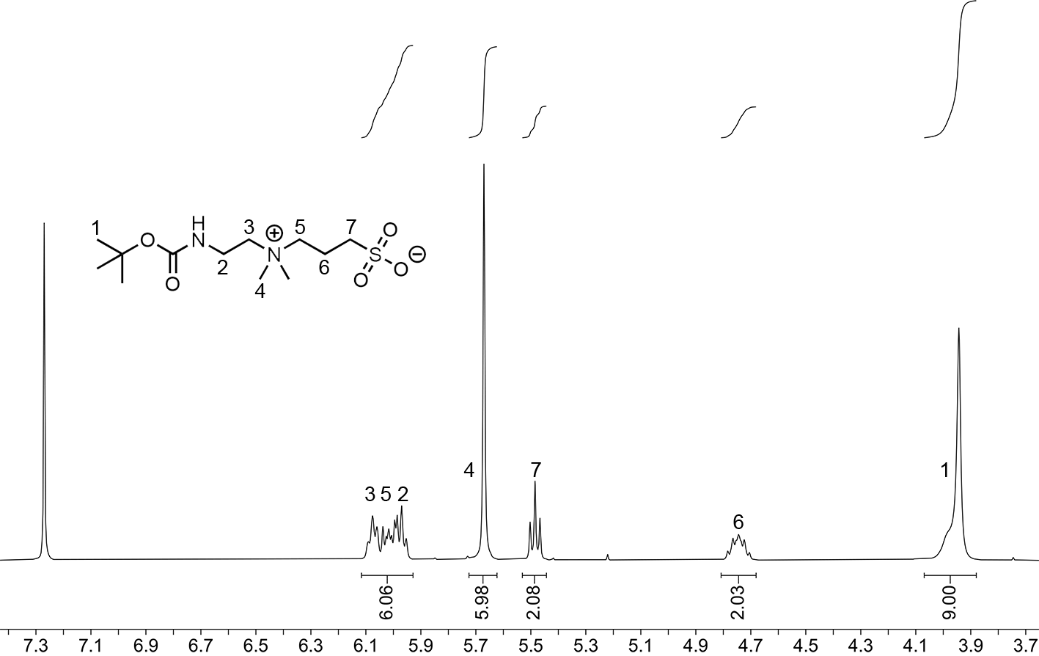


**Fig. S8** ^1^H NMR of compound **4**.


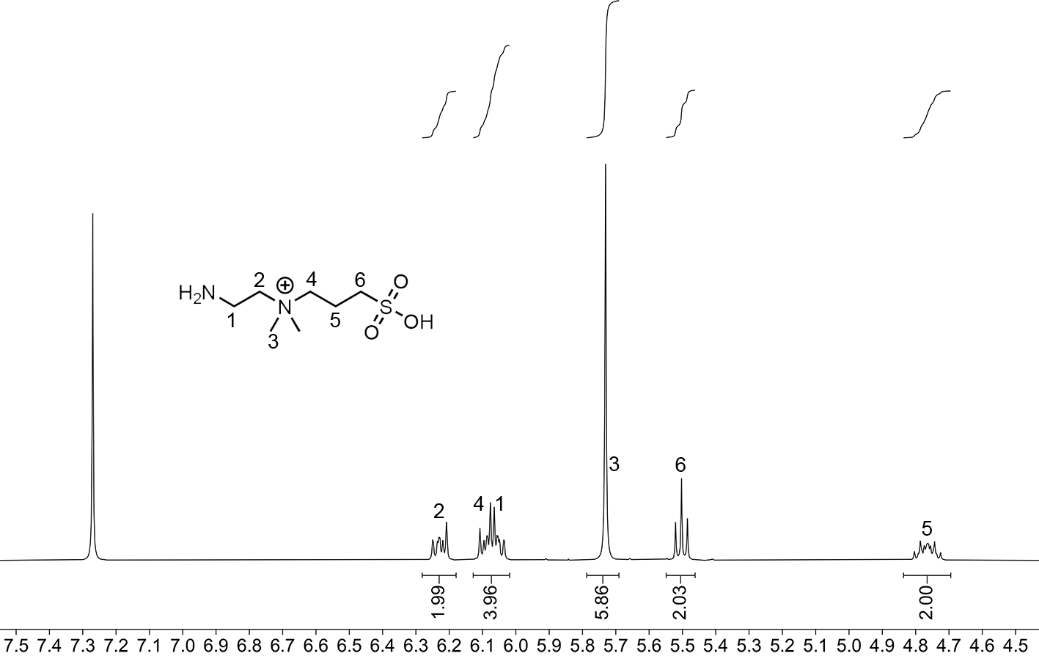


**Fig. S9** ^1^H NMR of compound **5**.


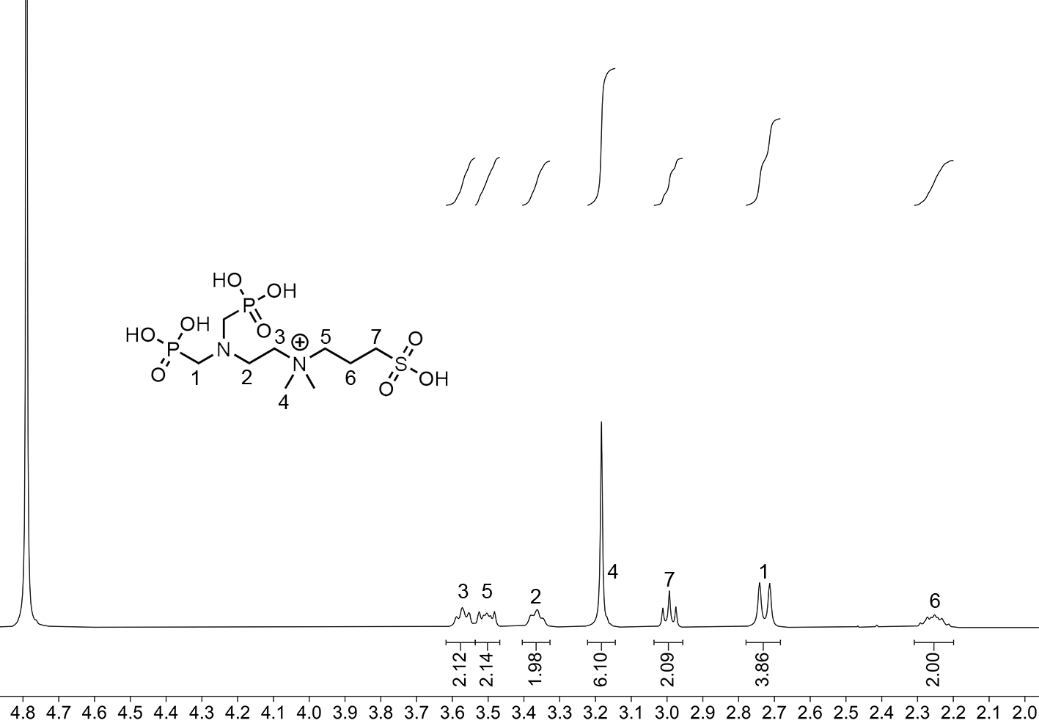


**Fig. S10** ^1^H NMR of DP-DMSA.


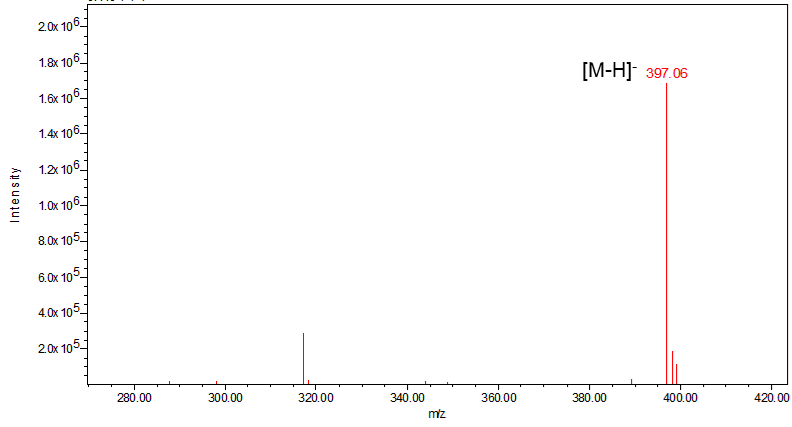


**Fig. S11** ESI-MS data of DP-DMSA.


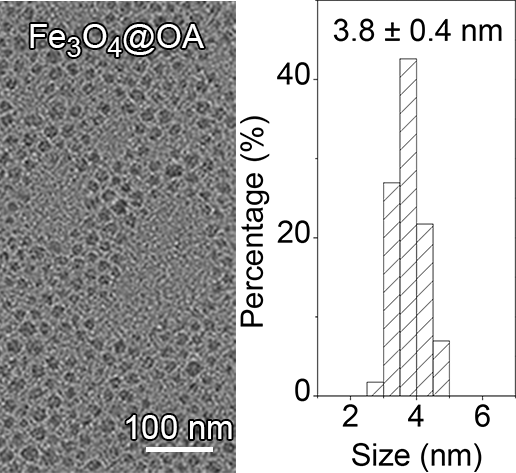


**Fig. S12** TEM image with corresponding size distribution of hydrophobic Fe_3_O_4_ nanoparticles.


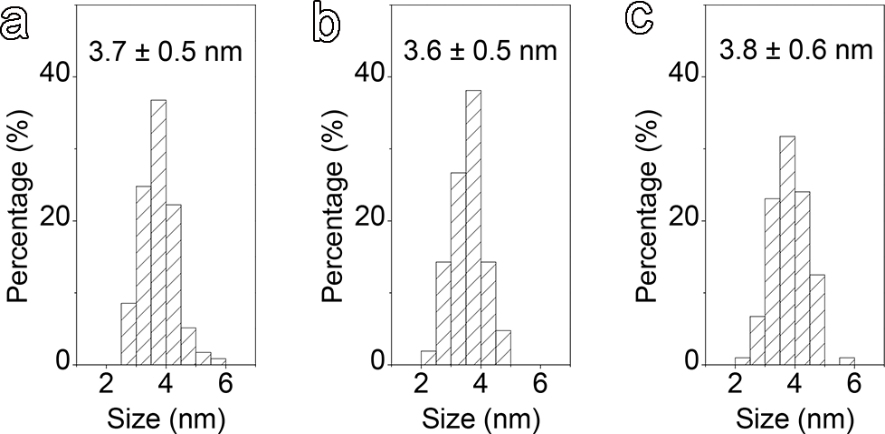


**Fig. S13** The corresponding size distribution of (a) Fe_3_O_4_@EACA, (b) Fe_3_O_4_@TMA, and (c) Fe_3_O_4_@DMSA nanoparticles, respectively.


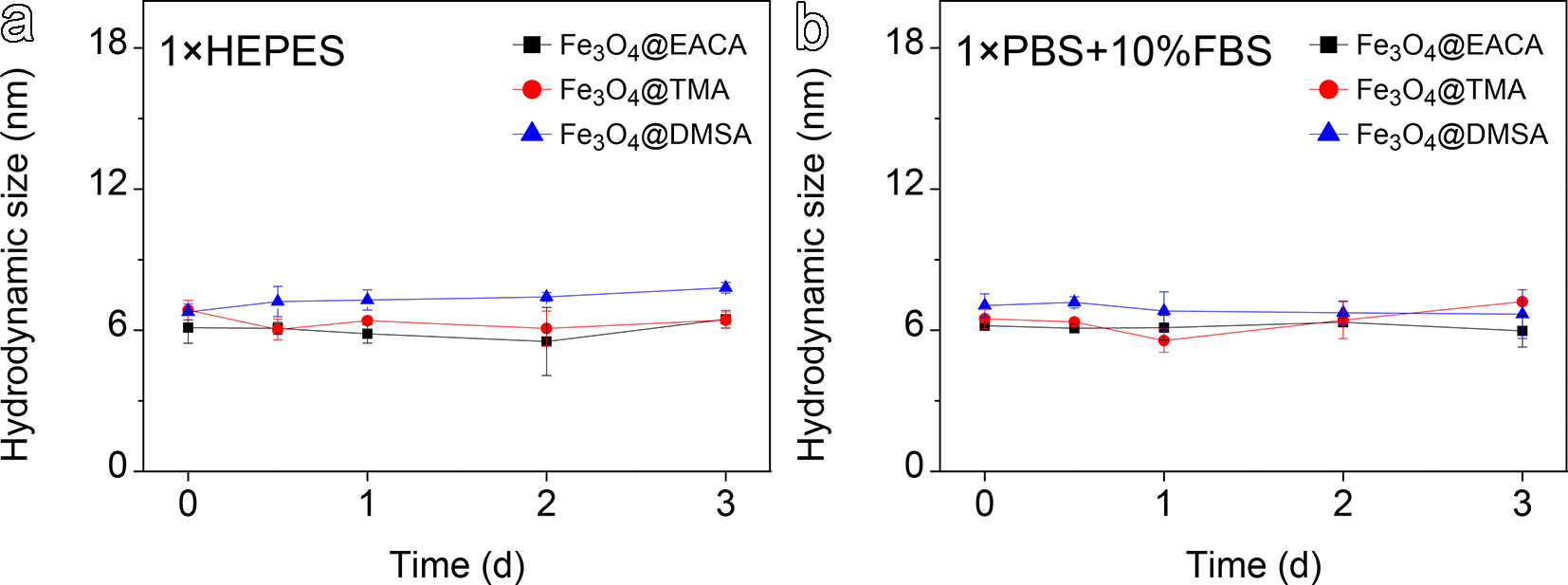


**Fig. S14** Hydrodynamic size changes of Fe_3_O_4_ nanoparticles incubated in (a) 1× HEPES and (b)1× PBS contain 10% FBS buffer.

**Table S1.** Ligand density on the surface of Fe_3_O_4_@EACA, Fe_3_O_4_@TMA, and Fe_3_O_4_@DMSA nanoparticles.

| Sample | Ligand Density  (number/nm^2^) |
| --- | --- |
| Fe_3_O_4_@EACA | 1.74 |
| Fe_3_O_4_@TMA | 1.43 |
| Fe_3_O_4_@DMSA | 1.00 |


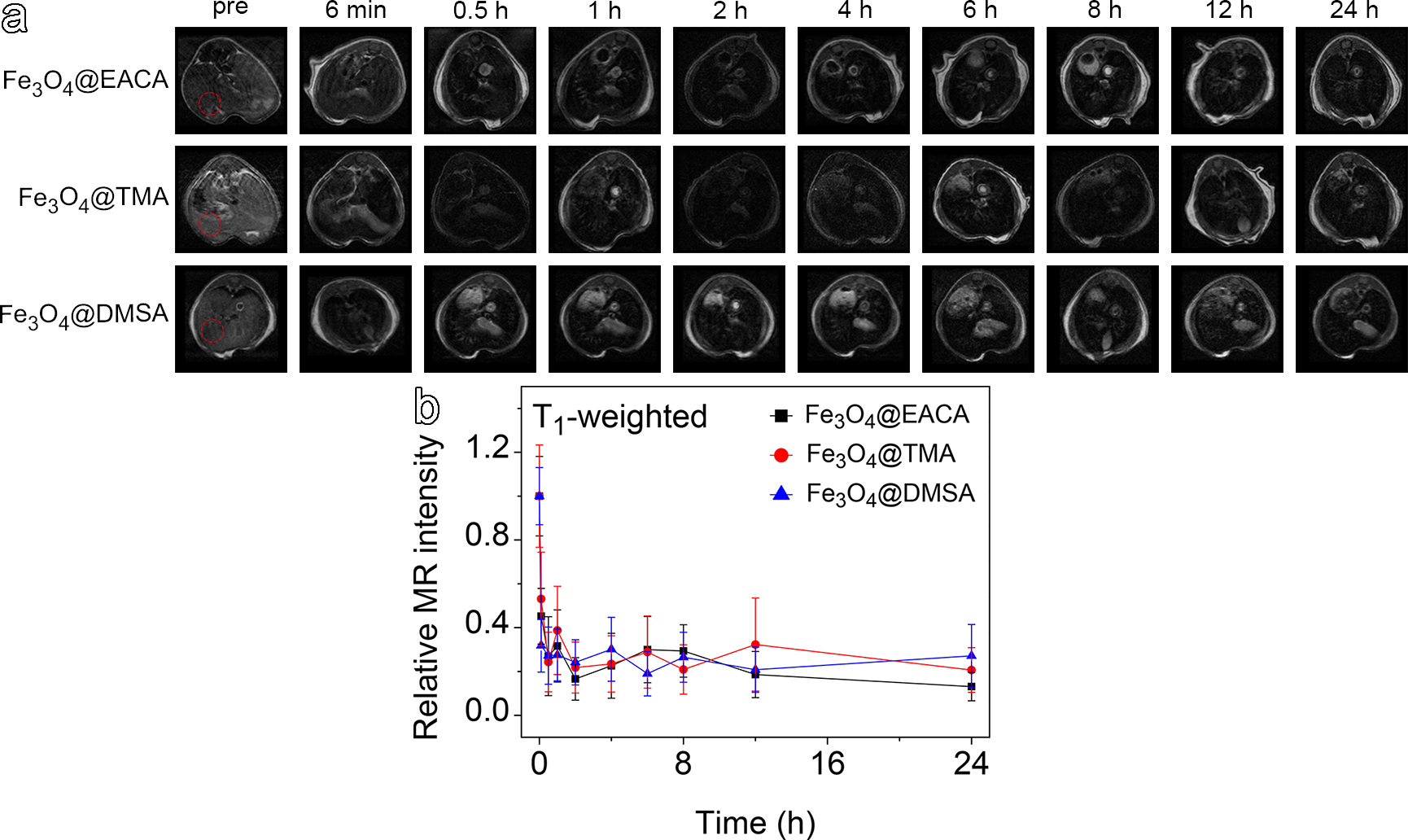


**Fig. S15** (a) T_1_-weighted MR images of liver at different time points following injection of ultrasmall Fe_3_O_4_ nanoparticles. (b) Temporal evolution of MR signals of the liver for T_1_-weighted imaging.


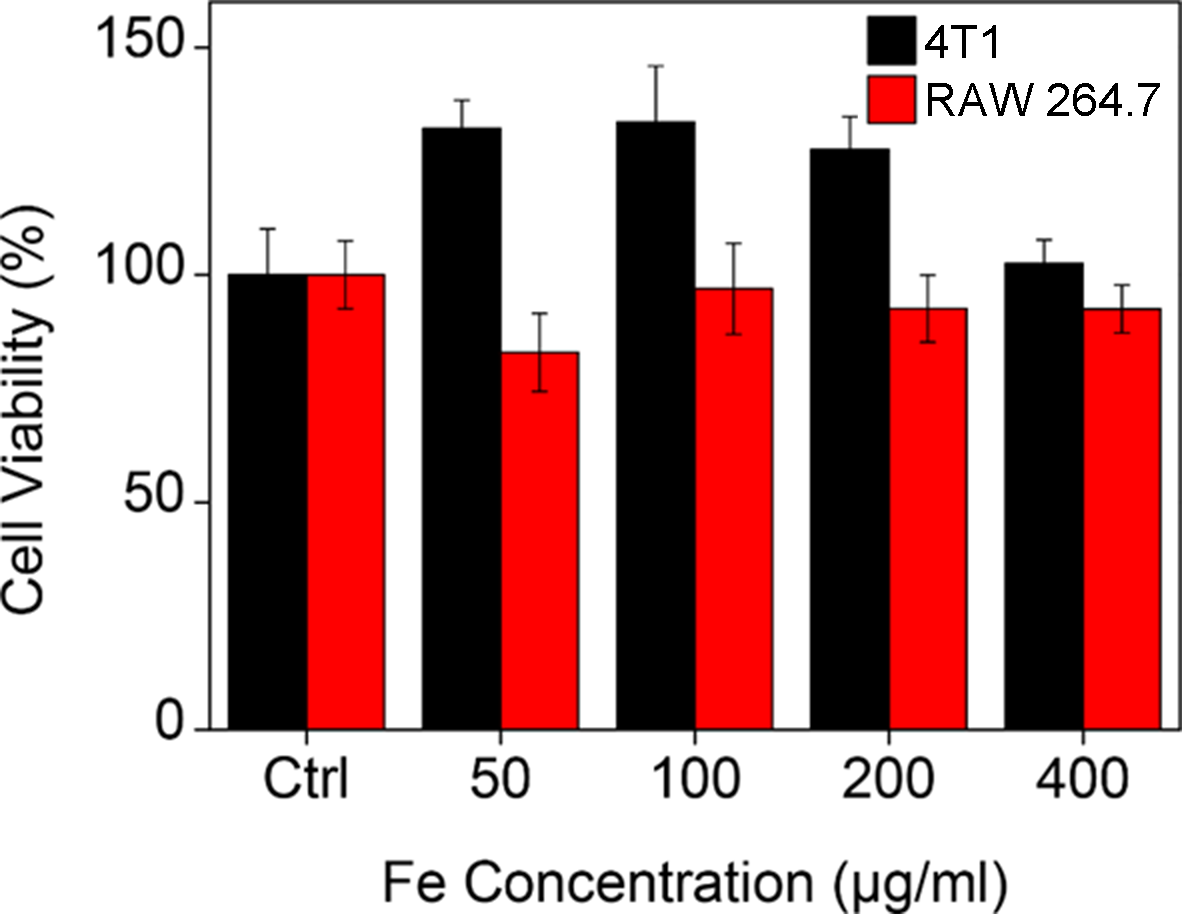


**Fig. S16** Cell viabilities of 4T1 and RAW 264.7 cells after incubation with Fe_3_O_4_@DMSA.


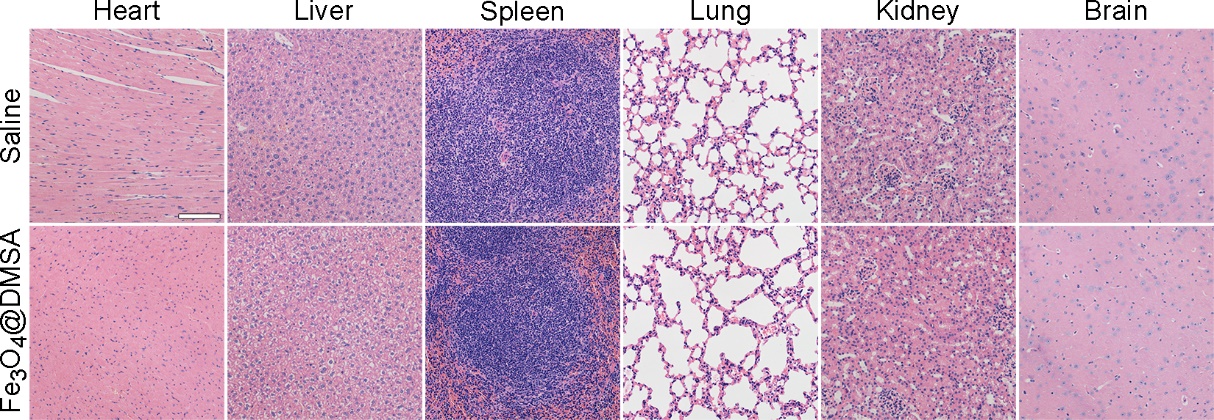


**Fig. S17** Images of H&E staining sections of tissues obtained from parts of the organs at 15 days post-injection of saline and Fe_3_O_4_@DMSA (scale bar = 200 μm).
